# Mental health profile and its relation with parental alcohol use problems and/or the experience of abuse in a sample of Moroccan high school students: an explorative study

**DOI:** 10.1186/s12991-019-0251-5

**Published:** 2019-12-19

**Authors:** Btissame Zouini, Anis Sfendla, Britt Hedman Ahlström, Meftaha Senhaji, Nóra Kerekes

**Affiliations:** 1 Department of Biology, Faculty of Sciences, Abdelmalek Essaadi University, Tetouan, Morocco; 2 Higher Institute of Nursing Professions and Health Techniques, Errachidia, Morocco; 3 Department of Health Sciences, University West, Trollhättan, Sweden

**Keywords:** Adolescents, Brief Symptom Inventory, Experience of abuse, Mental health, Morocco, Parental alcohol use problems

## Abstract

**Background:**

Studies on mental health are scarce from Arab countries, especially studies focusing on adolescents. In addition to the neurobiological and physiological changes that occur during adolescent development, psychological, societal and cultural influences have strong effects on adolescents’ behavior and on their somatic and mental health. The present study aimed (1) to describe the mental health profile, operationalized as psychological distress, of a sample of Moroccan adolescents, and (2) to investigate how specific psychosocial factors (parental alcohol use problems and the experience of physical and/or psychological abuse) may affect adolescents’ mental health.

**Methods:**

The sample included 375 adolescents from conveniently selected classes of four high schools in the city of Tetouan in Morocco. The participants responded to an anonymous survey containing, beside other inventories, the Brief Symptom Inventory (BSI) and identified those reporting parental alcohol use problems and/or the previous experience of abuse. The sample characteristics were defined using descriptive statistics. The effects of the defined psychosocial factors were identified using the Kruskal–Wallis test, followed by the post hoc Fisher’s least significant difference test.

**Results:**

The most common problems found in high school students from an urban region of Morocco were memory problems, concentration difficulties, restlessness, fear, nervosity and feelings of inadequacy during interpersonal interactions. The female students reported significantly higher psychological distress levels when compared to the male students (*p* < 0.001). The adolescents reporting parental alcohol use problems and the experience of physical/psychological abuse showed significantly higher levels of psychological distress (*p* = 0.02), especially symptoms of somatization (*p* < 0.001), hostility (*p* = 0.005) and anxiety (*p* = 0.01), than those not reporting any of these psychosocial factors.

**Conclusion:**

The mental health profile of female adolescents from an urban area of Morocco is worse than that of their male fellow students. Adolescents reporting parental alcohol use problems and/or the experience of physical/psychological abuse need synchronized support from social- and healthcare services.

## Introduction

Adolescence is a period of life with specific health and developmental needs. It is one of the most rapid phases of human development, during which adolescents form their identity, learn how to control their emotions and relationships, and acquire various abilities and attributes, such as self-reliance, work orientation, social commitment, openness to sociopolitical change, and tolerance of individual and cultural differences [1], as well as skills that can be important for their well-being [2]. In the adolescent brain there is a heightened responsiveness to stimuli (positive and negative) and to socioemotional contexts, while impulse control is still relatively immature [3]. Therefore, this period of life is characterized by various vulnerabilities for the adolescent. The vulnerabilities include both risky behaviors, such as drug abuse or violence, and vulnerability to psychiatric problems [4, 5]. In Morocco, 8.9% of the population, almost three million individuals, are aged between 15 and 19 years [6]. According to a report by the Moroccan Ministry of Health [7], almost every second adolescent (48.9%) has a problem with insomnia, anxiety and/or depression. One in five children and adolescents in Morocco suffers from a mental disorder; in half of these cases the age of onset was 14. These figures can explain why mental health recently emerged as one of Morocco’s main health objectives [7, 8].

The lifestyle of an adolescent’s parents has a significant effect on his or her well-being [9]. Adolescents whose parents have alcohol use problems are exposed to an increased risk of developing alcohol or drug use problems themselves, as well as an increased risk of encountering serious psychological problems [10]. Indeed, these adolescents report higher levels of depression, anxiety and/or stress than do adolescents whose parents do not have alcohol use problems [11, 12].

Furthermore, the experience of physical and/or psychological abuse during adolescence may be associated with general psychological distress, conduct problems, and aggression [13–17], as well as with increased risk of severe substance abuse problems [13, 18]. Such abuse is often combined in adolescents with lower self-esteem and higher levels of major depression, anxiety disorder and self-harm behavior [13, 19, 20]. In addition, adolescents with a combined history of physical and sexual abuse show higher scores on dissociation and somatization problems than do adolescents without any history of such abuse [21, 22].

The present study describes the psychological distress level in a sample of adolescents from an urban area in Morocco (the city of Tetouan), and investigates the relation between negative psychosocial factors (parental alcohol use problems and/or the experience of physical/psychological abuse) and the adolescents’ mental health.

## Methods

### Study population

This study was carried out within the framework of the “Mental and Somatic Health without borders” (MeSHe) project. MeSHe is an international project focusing on culture-specific patterns of mental health profiles coupled to substance abuse and aggressive antisocial behavior in adolescents [23]. The study population included students (*N* = 375; 170 males and 205 females) from conveniently selected tenth-, eleventh- and twelfth-grade classes at four high schools in the city of Tetouan, Morocco. With the authorization of the four school directors, data collection was done in the course of the 2014/15 school year. In total, the four high schools had 97 classes. Two classes from each grade and from each school were selected to participate in the study. In the 24 conveniently selected classes there were 876 students enrolled, of which 375 (43%) participated and completed the survey, representing 2.42% of the entire high school student population in the city of Tetouan (*N* = 15,506 students spread across 17 high schools). The age range in the study population was 15 to 18 years old and the mean age was 16.56 (SD = 1.04) years.

### Measures

#### MeSHe background inventory

The MeSHe survey includes, beside a list of validated measures of drug and alcohol abuse, antisocial aggressive behavior, and psychological distress, a detailed background questionnaire assessing the respondent’s age, gender, and presence of clinically diagnosed physical health problems. The background section of the questionnaire also contains items about environmental, psychosocial factors. Two of these items are stated as follows: “Have you ever been physically and/or psychologically abused?” and “Do you have a parent who has problems with alcohol?”. Based on their answers to these two questions, the responding adolescents in this study were classified into either of four groups: Adolescents not reporting having parental alcohol use problems nor the experience of being abused (comparison group: CG) (*n* = 250); adolescents reporting parental alcohol use problems (PAP) (*n* = 33); adolescents reporting the experience of physical and/or psychological abuse (PPA) (*n* = 55); and adolescents reporting both parental alcohol use problems and the experience of physical and/or psychological abuse (PAP + PPA) (*n* = 19). Of the 375 students participating in this study, 18 did not answer one or both of these questions, and were consequently excluded from the comparison between groups.

#### Brief Symptom Inventory

The MeSHe survey includes the Brief Symptom Inventory (BSI), which is a brief form of the Symptom Checklist Revised (SCL-90-R) [24, 25], a self-reporting inventory developed to measure an individual’s level of psychological distress [26]. The BSI has been translated into over 24 languages, including Arabic [27]. In this study, the responding adolescents were asked to rate the general influence of each item on their well-being over the past year.

The BSI contains 53 items, each of which is rated on a five-point Likert scale ranging from 0 (“not at all”) to 4 (“extremely”). Nine primary symptom dimensions of psychological distress are assessed within the BSI, namely somatization (SOM), obsessive compulsiveness (OBS), interpersonal sensitivity (INS), depression (DEP), anxiety (ANX), hostility (HOS), phobic anxiety (PHOB), paranoid ideation (PAR), and psychoticism (PSY). In addition to the nine symptom dimensions, the Global Severity Index (GSI), an indicator of the current overall level of distress, can be calculated [28].

The BSI can also be used in non-psychiatric adult populations [29, 30] and adolescents [31]. The BSI’s acceptable or good validity and its reliability measures have been established [32, 33]. In this study, the internal reliability of the primary symptom dimensions and the GSI was tested using internal consistency (Cronbach’s *α*); it was found to be acceptable for all dimensions and ranged from 0.71 (PSY) to 0.85 (DEP).

### Ethical considerations

The MeSHe survey was designed in accordance with the Helsinki Declaration [34] and its completion is voluntary and anonymous. The use of the survey was approved by the parent associations at each of the four high schools included in the study, by the Regional Directorate of the Ministry of National Education in Tetouan (with the registration number 85), responsible for managing and directing all matters concerning students from primary to high school education at Tetouan province, and by the Faculty of Science, University Abdelmalek Essaadi. Completion of the survey was considered as consent to participate.

All potential participants received a short written and oral presentation of the MeSHe project and its aims, and were given opportunity to discuss the project and their eventual participation with a responsible researcher; they were also offered the opportunity to leave the classroom if they did not want to participate in the study. The students were assured that their decision whether or not to participate would have no effect on their school record. The data from the responding students were collected on anonymous survey sheets.

### Statistical analysis

The sample characteristics were defined through descriptive statistics using SPSS version 21.0 (IBM). Because the scores of the BSI dimensions were not normally distributed in the study population, non-parametric statistical analyses were used. The Mann–Whitney *U* test was used to compare the scores of male and female students. The Kruskal–Wallis test was applied to compare the means ranks between the adolescents not reporting parental alcohol use problem nor the experience of abuse, the adolescents reporting parental alcohol use problems, the adolescents with experience of physical and/or psychological abuse, and the adolescents reporting both problems.

Post hoc (Fisher’s least significant difference) tests were applied for multiple testing regarding the differential interactions between the student groups. All the analyses were two-tailed and the significance level was defined at *p* < 0.05.

## Results

### Mental health of Moroccan adolescents from an urban area

Table 1 summarizes the mean values for each of the nine primary symptom dimensions of the Brief Symptom Inventory (BSI) and for the General Severity Index (GSI) in the Moroccan student sample. Generally, the responding Moroccan female students reported higher psychological distress when compared to their responding male fellow students. The female students scored significantly higher on all but one of the primary symptom dimensions; the exception being the “hostility” dimension where no significant difference could be measured between the genders. The generally higher psychological distress level in the female students is reflected also in their significantly higher GSI score.

**Table 1:** Self-reported psychiatric problems in the general population of Moroccan adolescents (*N* = 375)

BSI subscales	Moroccan adolescents *M* (SD)	Male (*n* = 144–169)^a^ *M* (SD)	Female (*n* = 163–199)^a^ *M* (SD)	*p*
Somatization	1.25 (0.83)	0.99 (0.78)	1.47 (0.81)	< 0.001
Obsessive compulsiveness	1.73 (0.83)	1.53 (0.82)	1.90 (0.81)	< 0.001
Psychoticism	1.39 (0.87)	1.26 (0.81)	1.50 (0.9)	0.02
Depression	1.22 (0.89)	1.07 (0.81)	1.34 (0.94)	0.01
Interpersonal sensitivity	1.52 (0.94)	1.17 (0.83)	1.81 (0.92)	< 0.001
Hostility	1.29 (0.82)	1.19 (0.76)	1.38 (0.86)	0.06
Phobic anxiety	1.16 (0.82)	0.96 (0.78)	1.34 (0.82)	< 0.001
Anxiety	1.58 (0.87)	1.24 (0.74)	1.84 (0.88)	< 0.001
Paranoid ideation	1.48 (0.83)	1.36 (0.81)	1.59 (0.83)	0.02
GSI	1.38 (0.68)	1.20 (0.67)	1.54 (0.65)	< 0.001

^a^The number of responses varies for the different subscales of the BSI

### Mental health of Moroccan adolescents reporting parental alcohol use problems or the experience of abuse

The majority of the responding high school students did not report the experience of physical nor psychological abuse (80.5%) and had no parent with alcohol use problems (86.4%). Nevertheless, a substantial number of adolescents reported the experience of physical and/or psychological abuse (14.7%) or the presence of at least one parent with problematic use of alcohol (8.8%). Of the students, 5.1% (*n* = 19) reported that they had experienced both physical and/or psychological abuse and a parent with alcohol use problems. There were significantly more male than female students reporting parental alcohol use problems (males: 11%; females: 7.8%; *p* = 0.002), or reporting both parental alcohol use problems and the experience of abuse (males: 9.8%; females: 1.6%; *p* = 0.03), whereas there were more female than male students reporting the experience of physical and/or psychological abuse, although this difference did not reach the significance level (females: 17.1%; males: 13.4%; *p* = 0.36). Because of the differences in the gender distribution in the responses to these questions, the level of psychological distress was analyzed separately.

In the PAP group (in both male and female students reporting parental alcohol use problems) none of the nine primary symptom dimension scores differed from the scores of the comparison group (CG).

The male students who reported both parental alcohol use problems and the experience of physical and/or psychological abuse (the PAP + PPA group) scored significantly higher than the male students not reporting any of these problems (CG) in the somatization (*p* < 0.001), the hostility (*p* = 0.005) and the anxiety (*p* = 0.01) primary symptom dimensions, as well as in the GSI (*p* = 0.01) (Table 2). The female students from the PPA group scored significantly higher in the somatization (*p* < 0.001), the obsessive-compulsiveness (*p* = 0.01), the psychoticism (*p* = 0.003), and the anxiety (*p* = 0.04) primary symptom dimensions compared to the female students in the CG; they also indicated significantly higher psychological distress levels in the depression (*p* = 0.01) and the hostility (*p* = 0.03) primary symptom dimensions, as well as in the GSI (*p* = 0.005), compared to the female students in both the CG and PAP groups (Table 3).

**Table 2:** Self-reported psychological distress in adolescent Moroccan males by psychosocial variable groups

	CG (*n* = 108)	PAP (*n* = 18)	PPA (*n* = 22)	PAP + PPA (*n* = 16)	Difference between groups		
	*M* (SD)	*M* (SD)	*M *(SD)	*M* (SD)	Test-stat (*H*)	*p* value	Post hoc
Somatization	0.90 (0.71)	0.94 (0.94)	1.07 (0.72)	1.63 (0.87)	10.42	0.02	CG < PAP + PPA* PAP < PAP + PPA* PPA < PAP + PPA*
Obsessive compulsiveness	1.47 (0.80)	1.65 (1.08)	1.53 (0.92)	1.75 (0.50)	1.73	0.63	NS
Psychoticism	1.17 (0.80)	1.3 (1.00)	1.46 (0.77)	1.55 (0.75)	4.85	0.18	NS
Depression	1.01 (0.82)	1.17 (1.00)	1.07 (0.83)	1.27 (0.49)	2.85	0.41	NS
Interpersonal sensitivity	1.13 (0.83)	1.15 (0.89)	1.00 (0.77)	1.55 (0.89)	3.93	0.27	PPA < PAP + PPA*
Hostility	1.10 (0.72)	1.21 (0.93)	1.18 (0.69)	1.67 (0.79)	7.26	0.06	CG < PAP + PPA* PPA < PAP + PPA*
Phobic anxiety	0.89 (0.74)	0.91 (0.83)	1.04 (0.81)	1.25 (0.90)	2.49	0.48	NS
Anxiety	1.16 (0.70)	1.22 (0.82)	1.25 (0.75)	1.69 (0.79)	5.93	0.11	CG < PAP + PPA*
Paranoid ideation	1.26 (0.73)	1.35 (0.89)	1.60 (1.00)	1.61 (0.86)	3.85	0.28	NS
GSI	1.12 (0.66)	1.28 (0.8)	1.19 (0.7)	1.56 (0.54)	6.28	0.10	CG < PAP + PPA*

*CG* comparison group, *PAP* adolescents reporting parental alcohol use problems, *PPA* adolescents reporting the experience of physical and/or psychological abuse, *PAP* + *PPA* adolescents reporting both parental alcohol use problems and the experience of physical and/or psychological abuse

*p < 0.05

**Table 3:** Self-reported psychological distress in adolescent Moroccan females by psychosocial variable groups

	CG (*n* = 142)	PAP (*n* = 15)	PPA (*n* = 33)	PAP + PPA (*n* = 3)	Difference between groups		
	(Min–Max)	(Min–Max)	(Min–Max)	(Min–Max)	Test-stat (*H*)	*p* value	Post hoc
Somatization	1.40 (0.83)	1.41 (0.77)	1.79 (0.81)	1.76 (0.44)	6.84	0.08	CG < PPA*
Obsessive compulsiveness	1.83 (0.81)	1.56 (0.85)	2.23 (0.74)	2.11 (0.92)	9.04	0.03	CG < PPA* PAP < PPA*
Psychoticism	1.40 (0.91)	1.31 (0.52)	1.92 (0.86)	0.80 (0.69)	12.23	0.01	CG < PPA* PAP < PPA*
Depression	1.22 (0.90)	1.12 (0.62)	1.87 (1.02)	0.94 (0.35)	12.37	0.01	CG < PPA** PAP < PPA*
Interpersonal sensitivity	1.73 (0.90)	1.49 (0.61)	2.03 (0.99)	2.25 (0.66)	6.13	0.10	NS
Hostility	1.27 (0.86)	1.24 (0.60)	1.83 (0.81)	1.40 (0.92)	12.39	0.01	CG < PPA** PAP < PPA*
Phobic anxiety	1.31 (0.83)	1.23 (0.82)	1.50 (0.84)	0.80 (0.00)	2.94	0.40	NS
Anxiety	1.77 (0.88)	1.59 (0.61)	2.12 (0.94)	2.22 (0.36)	4.99	0.17	CG < PPA*
Paranoid ideation	1.53 (0.84)	1.38 (0.58)	1.77 (0.87)	1.27 (0.46)	2.6	0.46	NS
GSI	1.45 (0.64)	1.37 (0.49)	1.92 (0.63)	1.52 (0.16)	13.13	< 0.01	CG < PPA** PAP < PPA*

*CG* comparison group, *PAP* adolescents reporting parental alcohol use problems, *PPA* adolescents reporting the experience of physical and/or psychological abuse, *PAP* + *PPA* adolescents reporting both parental alcohol use problems and the experience of physical and/or psychological abuse

*p < 0.05; **p < 0.001

## Discussion

### Mental health of Moroccan adolescents from an urban region

To the best of our knowledge, this study is the first to investigate the self-reported mental health of Moroccan adolescents. To measure mental health and symptomatic behavior, the well-known clinical instrument known as the Brief Symptom Inventory (BSI) [24, 26] was used. Although this instrument is most often used in clinical populations to measure treatment effects by assessing the patient’s feelings, it is also often used to measure mental health profiles in non-clinical populations as well. For instance, the BSI was used in a study assessing an adolescent sample of the general population in Israel [31]. Said study emphasized the need for culture-specific BSI norm-data in adolescent populations, as significant differences could be shown between American and Israeli adolescents’ scores in somatization, hostility, phobic anxiety, paranoid ideation and psychoticism, and between the two group’s overall distress score (GSI), with the American students reporting higher psychological distress levels [31, 35]. When we compare the levels of psychological distress reported in this study’s sample of Moroccan adolescents with the levels found in American and Israeli adolescents, we note that the Moroccan scores are the highest. However, this comparison should be handled with caution as the data from studies performed so far apart in time. To be able to establish culture-specific differences in adolescents’ psychological distress levels, we would need to compare our data with more recent studies. In the absence of such data, our only conclusion can be that adolescents living in a Moroccan urban area in 2014–2015 reported more symptoms and higher levels of psychological distress than did adolescents in developed or developing countries 10–20 years ago.

In line with previous studies [36–41], the scores of the male and female students in the present study differed significantly, with female students reporting more symptoms on all the BSI subscales, with the exception of hostility. Other studies have suggested that being female is associated with a higher prevalence of auditory verbal hallucinations, earlier onset of psychotic illness, greater affiliative need, and greater sensitivity to both conflict and rejection within interpersonal relationships [42–45]. These gender differences in the mental health profile of adolescents may be explained by gender-specific genetic factors [46–48], hormones [49], brain structure, function, circuitry, and pharmacokinetics [50, 51], but also by gender-specific exposure levels to the specific psychosocial environmental risk factors [50, 52].

### Mental health of Moroccan adolescents reporting parental alcohol use problems or reporting the experience of abuse

In the present study, we found that significantly more male than female students reported having parental alcohol use problems. These male students also reported high levels of psychological distress, manifested by their higher scores in the somatization, hostility, and anxiety dimensions of the BSI, when compared to their male classmates not reporting any of these problems (comparison group). These results may be explained by the fact that children of parents with alcohol use disorder exhibit a large probability of an earlier onset of substance use [11], of suffering from neglect [53], and of having cognitive deficit, behavioral and emotional difficulties, and psychosocial adjustment problems [54–57], and of having mental disorders [58]. Consequently, the presence of traumatic experiences in addition to the presence of a parent with problematic alcohol use may increase the risk of neurodevelopmental impairment [59]. Previous research has shown that abused children and adolescents are at higher risk of exhibiting aggression [60–62] and deficits in their emotional regulation [63]. Generally, emotional dysregulation is positively correlated with hostility [64] and represents a risk factor for internalizing problems, such as anxiety and somatic complaints [65].

Our results also show that the female students who reported the experience of physical/psychological abuse also reported higher levels of psychological distress, captured by significantly higher scores in the somatization, obsessive compulsiveness, psychoticism, anxiety, depression and hostility dimensions of the BSI, than did their female classmates in the comparison group. Previous research has found similar results, suggesting that the experience of abuse by female adolescents is significantly associated with anxiety, depression, dissociative disorder, and aggressive behavior [66–68]. Furthermore, previous research has shown that abuse of female adolescents may be associated with dysregulation of their emotional patterns [69], post-traumatic stress disorders [70], and low self-esteem [71], which are strongly linked to internalizing and externalizing behaviors, and negative affect such as depression, anxiety, hostility, somatization and psychoticism [72–74]. In our study population there were significantly more male than female students reporting both parental alcohol use problems and the experience of abuse. This may be an effect of the Islamic culture and education, which dissuades Arab female adolescents from reporting being physically or psychologically abused within their family, which may increase their feelings of solitude and isolation, which in turn are strongly associated with various psychiatric disorders [75–77].

## Conclusion

The present study provides the first insights into the self-reported mental health profiles of Moroccan adolescents and underlines the need for new assessments in order to make international comparisons.

The study provides evidence that female high school students report higher psychological distress levels compared to their male classmates. Furthermore, the study confirms the serious and diverse negative relation between parental alcohol use problems and/or of the experience of physical/psychological abuse and the mental health of adolescents. Interventions and support for these adolescents from both social- and healthcare organizations are warranted.

## Limitations

The study had a cross-sectional design not allowing any causality analyses. Despite including data from almost 400 adolescents, the study’s generalizability is limited; the study population represented only a fraction of all adolescents in Morocco and was selected from schools in only one city. The study’s use of self-reporting entails well-known limitations, namely that self-report questionnaires depend on the respondent’s ability and willingness to remember and answer truthfully; responses may be distorted by social desirability and recall biases [78].

The assessment of the physical and/or psychological abuse did not include the degree or frequency of abuse, any associated disability, or information on the specific type of abuse experienced by the adolescent. It is noteworthy that, based on this limitation and other recognized limitations of the assessed data, an improved version of the survey has been developed for future use in the MeSHe project.

## Data Availability

The data sets used and/or analyzed in the course of this study are available from the corresponding author on reasonable request.

## Abbreviations

ANX: anxiety
BSI: Brief Symptom Inventory
CG: comparison group
DEP: depression
GSI: global severity index
HOS: hostility
INS: interpersonal sensitivity
MeSHe project: Mental and Somatic Health without borders project
OBS: obsessive compulsiveness
PAP: adolescents reporting having parents with alcohol problems
PAP + PPA: adolescents reporting both parental alcohol use problems and the experience of physical and/or psychological abuse
PAR: paranoid ideation
PHOB: phobic anxiety
PPA: adolescents reporting the experience of physical and/or psychological abuse
PSY: psychoticism
SOM: somatization

## References

[1] Greenberger E (1984). Defining psychosocial maturity in adolescence. Adv Child Behav Anal Ther.

[2] World Health Organisation (WHO). Maternal, newborn, child and adolescent health. https://www.who.int/maternal_child_adolescent/topics/adolescence/development/en/ (1984). Accessed 20 Mar 2016.

[3] Casey BJ, Jones RM, Hare TA (2008). The adolescent brain. Ann N Y Acad Sci..

[4] Welsh JW, Knight JR, Hou SS, Malowney M, Schram P, Sherritt L (2017). Association between substance use diagnoses and psychiatric disorders in an adolescent and young adult clinic-based population. J Adolesc Health..

[5] Schulte-Körne G (2016). Mental health problems in a school setting in children and adolescents. Dtsch Arztebl Int..

[6] High Commission for Planning (HCP). Démographie-Maroc. https://rgphentableaux.hcp.ma/Default1/ (2014). Accessed 21 Mar 2016.

[7] Moroccan Ministry of Health. La 2ème rencontre nationale sur la santé scolaire et universitaire et la promotion de la santé des jeunes. https://www.sante.gov.ma/Documents/Actualites/disscours-03-2014fr.pdf (2014). Accessed 20 Mar 2016.

[8] World Health Organisation (WHO). Stratégie de cooperation OMS-Maroc 2017–2021. https://extranet.who.int/iris/restricted/bitstream/10665/254588/5/CCS_Maroc_2016_fr_19364.pdf?ua=1 (2016). Accessed 21 Mar 2016.

[9] Milevsky A, Schlechter M, Netter S, Keehn D (2007). Maternal and paternal parenting styles in adolescents: associations with self-esteem, depression and life-satisfaction. J Child Fam Stud..

[10] Windle M (1996). Effect of parental drinking on adolescents. Alcohol Res Health..

[11] Chassin L, Rogosch F, Barrera M (1991). Substance use and symptomatology among adolescent children of alcoholics. J Abnorm Psychol..

[12] Stanley S, Vanitha C (2008). Psychosocial correlates in adolescent children of alcoholics—implications for intervention. IJPR..

[13] Fergusson DM, Horwood LJ, Lynskey MT (1996). Childhood sexual abuse and psychiatric disorder in young adulthood: II. Psychiatric outcomes of childhood sexual abuse. Am Acad Child Adolesc Psychiatry..

[14] Shapero BG, Black SK, Liu RT, Klugman J, Bender RE, Abramson LY (2014). Stressful life events and depression symptoms: the effect of childhood emotional abuse on stress reactivity. J Clin Psychol..

[15] Landolt MA, Schnyder U, Maier T, Mohler-Kuo M (2016). The harm of contact and non-contact sexual abuse: health-related quality of life and mental health in a population sample of Swiss adolescents. Psychother Psychosom..

[16] Tlapek SM, Auslander W, Edmond T, Gerke D, Schrag RV, Threlfall J (2017). The moderating role of resiliency on the negative effects of childhood abuse for adolescent girls involved in child welfare. Child Youth Serv Rev..

[17] Alizzy A, Calvete E, Bushman BJ (2017). Associations between experiencing and witnessing physical and psychological abuse and internalizing and externalizing problems in Yemeni children. J Fam Violence..

[18] Sartor CE, Waldron M, Duncan AE, Grant JD, McCutcheon VV, Nelson EC (2013). Childhood sexual abuse and early substance use in adolescent girls: the role of familial influences. Addiction.

[19] Kim JY, Lee K (2015). Effect of adolescents’ abuse experience on suicidal ideation: focused on moderated mediation effect of self-esteem on depression and anxiety. J Korean Acad Nurs..

[20] Lereya ST, Copeland WE, Costello EJ, Wolke D (2015). Adult mental health consequences of peer bullying and maltreatment in childhood: two cohorts in two countries. Lancet Psychiatry..

[21] Atlas JA, Wolfson MA, Lipschitz DS (1995). Dissociation and somatization in adolescent inpatients with and without history of abuse. Psychol Rep..

[22] Marquis C, Vabres N, Caldagues E, Bonnot E (2016). Clinique des troubles somatoformes chez les adolescents maltraités. Presse Med.

[23] Mental and Somatic Health Without Borders (MeSHe) Project. https://meshe.se/. Accessed 21 Mar 2016.

[24] Derogatis LR. Brief Symptom Inventory. Baltimore, MD: Clinical Psychometric Research. 1975. https://hazards.colorado.edu/nhcdata/chernobyl/ChData/ScalesInstruments/Scales%20and%20Indices/Scale%20Construction%20Instructions/BSI.pdf. Accessed 15 Jan 2017.

[25] Derogatis LR (1977). The SCL-R-90 manual I: scoring, administration and procedures for the SCL-90.

[26] Derogatis LR, Spencer PM (1982). The Brief Symptom Inventory (BSI): administration, and procedures manual-I.

[27] Abdallah T (1998). The satisfaction with life scale (SWLS): psychometric properties in an Arabic-speaking sample. Int J Adolesc Youth..

[28] Derogatis LR, Melisaratos N (1983). The Brief Symptom Inventory: an introductory report. Psychol Med..

[29] Aroian KJ, Patsdaughter CA, Levin A, Gianan ME (1995). Use of the Brief Symptom Inventory to assess psychological distress in three immigrant groups. Int J Soc Psychiatry..

[30] Gilbar O, Ben-Zur H (2002). Adult Israeli community norms for the Brief Symptom Inventory (BSI). Int J Stress Manag..

[31] Canetti L, Shalev AY, De-Nour AK (1994). Israeli adolescents’ norms of the Brief Symptom Inventory (BSI). Isr J Psychiatry Relat Sci..

[32] Derogatis LR, Cleary PA (1977). Confirmation of the dimensional structure of the SCL-90: a study in construct validation. J Clin Psychol..

[33] Hoe M, Brekke J (2009). Testing the cross-ethnic construct validity of the Brief Symptom Inventory. Res Soc Work Pract..

[34] World Medical Association (WMA). Declaration of Helsinki—Ethical principles for medical research involving human subjects, 64th WMA General Assembly, Fortaleza, Brazil, October 2013. https://www.wma.net/policies-post/wma-declaration-of-helsinki-ethical-principles-for-medical-research-involving-human-subjects/ (1964). Accessed 03 May 2018.

[35] Derogatis LR (1992). SCL-90-R: administration, scoring & procedures manual-II, for the R (Revised) version and other instruments of the psychopathology rating scale series.

[36] Garber J, Walker LS, Zeman J (1991). Somatization symptoms in a community sample of children and adolescents: further validation of the Children’s Somatization Inventory. Psychol Assess..

[37] Hankin BL (2009). Development of sex differences in depressive and co-occurring anxious symptoms during adolescence: Descriptive trajectories and potential explanations in a multiwave prospective study. J Clin Child Adolesc Psychol..

[38] Wigman JT, Vollebergh WA, Raaijmakers QA, Iedema J, Van Dorsselaer S, Ormel J (2009). The structure of the extended psychosis phenotype in early adolescence—a cross-sample replication. Schizophr Bull..

[39] Park JH, Bang YR, Kim CK (2014). Sex and age differences in psychiatric disorders among children and adolescents: high-risk students study. Psychiatry Investig..

[40] Vivan ADS, Rodrigues L, Wendt G, Bicca MG, Braga DT, Cordioli AV (2014). Obsessive-compulsive symptoms and obsessive-compulsive disorder in adolescents: a population-based study. Rev Bras Psiquiatr..

[41] Ohannessian CM, Milan S, Vannucci A (2017). Gender differences in anxiety trajectories from middle to late adolescence. J Youth Adolesc..

[42] Morokuma Y, Endo K, Nishida A, Yamasaki S, Ando S, Morimoto Y (2017). Sex differences in auditory verbal hallucinations in early, middle and late adolescence: results from a survey of 17,451 Japanese students aged 12–18 years. BMJ Open..

[43] Galdos PM, Van OJJ, Murray RM (1993). Puberty and the onset of psychosis. Schizophr Res..

[44] Cyranowski JM, Frank E, Young E, Shear MK (2000). Adolescent onset of the gender difference in lifetime rates of major depression: a theoretical model. Arch Gen Psychiatry..

[45] Prinstein MJ, Aikins JW (2004). Cognitive moderators of the longitudinal association between peer rejection and adolescent depressive symptoms. J Abnorm Child Psychol..

[46] Payer B, Lee JT (2008). X chromosome dosage compensation: how mammals keep the balance. Annu Rev Genet..

[47] Kang HJ, Kawasawa YI, Cheng F, Zhu Y, Xu X, Li M (2011). Spatio-temporal transcriptome of the human brain. Nature.

[48] Qin W, Liu C, Sodhi M, Lu H (2016). Meta-analysis of sex differences in gene expression in schizophrenia. BMC Syst Biol.

[49] Seeman MV (1997). Psychopathology in women and men: focus on female hormones. Am J Psychiatry..

[50] Zahn-Waxler C, Shirtcliff EA, Marceau K (2008). Disorders of childhood and adolescence: gender and psychopathology. Annu Rev Clin Psychol..

[51] Ruigrok AN, Salimi-Khorshidi G, Lai MC, Baron-Cohen S, Lombardo MV, Tait RJ (2014). A meta-analysis of sex differences in human brain structure. Neurosci Biobehav Rev..

[52] Biederman J, Faraone SV, Monuteaux MC (2002). Differential effect of environmental adversity by gender: Rutter’s index of adversity in a group of boys and girls with and without ADHD. Am J Psychiatry..

[53] Dube SR, Anda RF, Felitti VJ, Croft JB, Edwards VJ, Giles WH (2001). Growing up with parental alcohol abuse: exposure to childhood abuse, neglect, and household dysfunction. Child Abuse Negl.

[54] Bennett LA, Wolin SJ, Reiss D (1988). Cognitive, behavioral, and emotional problems among school-age children of alcoholic parents. Am J Psychiatry..

[55] Hussong AM, Zucker RA, Wong MM, Fitzgerald HE, Puttler LI (2005). Social competence in children of alcoholic parents over time. Dev Psychol..

[56] Greenbaum RL, Stevens SA, Nash K, Koren G, Rovet J (2009). Social cognitive and emotion processing abilities of children with fetal alcohol spectrum disorders: a comparison with attention deficit hyperactivity disorder. Alcohol Clin Exp Res..

[57] Kingdon D, Cardoso C, McGrath JJ (2015). Research review: executive function deficits in fetal alcohol spectrum disorders and attention-deficit/hyperactivity disorder—a meta-analysis. J Child Psychol Psychiatry..

[58] Anda RF, Whitfield CL, Felitti VJ, Chapman D, Edwards VJ, Dube SR (2002). Adverse childhood experiences, alcoholic parents, and later risk of alcoholism and depression. Psychiatr Serv..

[59] Price A, Cook PA, Norgate S, Mukherjee R (2017). Prenatal alcohol exposure and traumatic childhood experiences: a systematic review. Neurosci Biobehav Rev..

[60] Holmes MR, Yoon S, Voith LA, Kobulsky JM, Steigerwald S (2015). Resilience in physically abused children: protective factors for aggression. Behav Sci..

[61] Shields A, Cicchetti D (1998). Reactive aggression among maltreated children: the contributions of attention and emotion dysregulation. J Clin Child Psychol..

[62] Zouini B, Senhaji M, Kerekes N (2019). Self-reported aggressive and antisocial behaviors in Moroccan high school students. Psihologija.

[63] Tatnell R, Hasking P, Newman L, Taffe J, Martin G (2016). Attachment, emotion regulation, childhood abuse and assault: examining predictors of NSSI among adolescents. Arch Suicide Res..

[64] Mitrofan N, Ciulvica C (2012). Anger and hostility as indicators of emotion regulation and of the life satisfaction at the beginning and the ending period of the adolescence. Procedia Soc Behav Sci..

[65] McLaughlin KA, Hatzenbuehler ML, Mennin DS, Nolen-Hoeksema S (2011). Emotion dysregulation and adolescent psychopathology: a prospective study. Behav Res Ther..

[66] Halpern CT, Tucker CM, Bengtson A, Kupper LL, McLean SA, Martin SL (2013). Somatic symptoms among US adolescent females: associations with sexual and physical violence exposure. Matern Child Health J..

[67] Jeffrey TB, Jeffrey LK (1991). Psychologic aspects of sexual abuse in adolescence. Curr Opin Obstet Gynecol..

[68] Auslander W, Sterzing P, Threlfall J, Gerke D, Edmond T (2016). Childhood abuse and aggression in adolescent girls involved in child welfare: the role of depression and posttraumatic stress. J Child Adolesc Trauma..

[69] Maughan A, Cicchetti D (2002). Impact of child maltreatment and interadult violence on children’s emotion regulation abilities and socioemotional adjustment. Child Dev..

[70] Hébert M, Lavoie F, Blais M (2014). Post-Traumatic Stress Disorder/PTSD in adolescent victims of sexual abuse: resilience and social support as protection factors. Cien Saude Colet..

[71] Kim BN, Park S, Park MH (2017). The relationship of sexual abuse with self-esteem, depression, and problematic internet use in Korean adolescents. Psychiatry Investig..

[72] Buckholdt KE, Parra GR, Jobe-Shields L (2014). Intergenerational transmission of emotion dysregulation through parental invalidation of emotions: implications for adolescent internalizing and externalizing behaviors. J Child Fam Stud..

[73] Bekh Bradley D, DeFife JA, Guarnaccia C, Phifer MJ, Fani MN, Ressler KJ (2011). Emotion dysregulation and negative affect: association with psychiatric symptoms. J Clin Psychiatry..

[74] Neiss MB, Stevenson J, Legrand LN, Iacono WG, Sedikides C (2009). Self-esteem, negative emotionality, and depression as a common temperamental core: a study of mid-adolescent twin girls. J Pers..

[75] Peter D. Self-compassion, self-criticism, parent-child attachment moderate the relation between anxious solitude and psychosocial adjustment in early adolescence. Melbourne School of Psychological Sciences. Doctoral dissertation. 2016.

[76] Matthews T, Danese A, Wertz J, Odgers CL, Ambler A, Moffitt TE (2016). Social isolation, loneliness and depression in young adulthood: a behavioural genetic analysis. Soc Psychiatry Psychiatr Epidemiol..

[77] Hall-Lande JA, Eisenberg ME, Christenson SL, Neumark-Sztainer D (2007). Social isolation, psychological health, and protective factors in adolescence. Adolescence..

[78] Lajunen T, Özkan T, Bryan EP (2011). Self-report instruments and methods. Handbook of traffic psychology.

